# Caregiver Burden in Alcohol Dependence Syndrome

**DOI:** 10.1155/2017/8934712

**Published:** 2017-05-21

**Authors:** Ramanujam Vaishnavi, Murugan Selvaraj Karthik, Ramasamy Balakrishnan, Ramanathan Sathianathan

**Affiliations:** Department of Psychiatry, Sri Ramachandra Medical College and Research Institute, Sri Ramachandra University, Porur, Chennai 600116, India

## Abstract

**Background:**

Alcoholism is a major threat to the individual as well as the society and the maximum burden of the illness is borne by the family.

**Aim:**

The study is aimed at assessing the pattern of burden on the caregivers of alcohol dependent patients and at assessing the relationship between the severity of dependence and the burden on caregivers.

**Settings and Design:**

Cross-sectional descriptive study conducted in the Department of Psychiatry, Sri Ramachandra Medical College and Research Institute.

**Materials and Methods:**

A cross-sectional assessment was done in 200 patients with alcohol dependence and their caregivers. The severity of dependence and the pattern of burden on caregivers were assessed.

**Statistical Analysis:**

The data thus collected was analyzed using SPSS version 20.

**Results:**

The study demonstrates that caregivers of alcohol dependent patients reported significant objective burden and subjective burden. Furthermore, the severity of alcohol dependence and the domains of burden such as financial burden, disruption of family interaction, and disruption of family routine activities were positively correlated with high level of significance.

**Conclusion:**

The current study has illustrated that all the caregivers experienced significant amount of burden which has to be addressed for better treatment outcome of the patients.

## 1. Introduction

Family plays a key role in the care of patients with mental illnesses. This is especially very true in India because of various factors like the tradition of interdependence, the concern for the family, and the lack of sufficient mental health professionals [1]. Alcohol dependence has been a major social and personal threat in most countries. According to Global Status Report on Alcohol, Alcohol Use Disorders (AUDs) account for 1.4 per cent of the global disease burden [2]. A nationwide Indian study on alcohol and drug abuse by Sarkar et al. estimated the prevalence of alcohol use as 21.4% [3]. A study from southern rural India showed that 14.2% of the population surveyed had hazardous alcohol use on the Alcohol Use Disorder Identification Test (AUDIT) [4]. Another study from the tertiary care hospital at a rural district of Southern India reported that 17.6% of admitted patients had hazardous alcohol use [5]. Alcohol dependence is considered as a “family disease.” Alcohol dependence affects the individual as well as those around them in terms of occupational and social dysfunction, physical and emotional distress, and financial burden which has a serious impact on the lives of the significant others [6]. An earlier study from India comparing the family burden of patients with schizophrenia, alcohol dependence, and opioid dependence by using the Family Burden Interview Schedule (FBIS) showed moderate to severe burden in all the three groups [7]. Another study assessed the severity of burden in wives of opioid dependence patients and reported severe burden in both objective and subjective scales [8]. A study from Nepal among intravenous drug users and alcohol dependent patients found increased caregiver burden in both the groups; however the burden was more with intravenous drug users than alcohol dependent patients. The study also reported that the spouses of both alcohol dependent patients and also intravenous drug abusers exhibited more tolerance and less perceived burden towards the substance use when compared to the other family members like parents, children, and siblings [9]. A study from Chandigarh which assessed the family burden using FBIS in 120 subjects of alcohol and/or opioid dependence reported that almost all (95–100%) caregivers had severe burden [10]. Although such studies have been conducted in the Northern part of India, the number of such studies is very limited in Southern India. And as alcohol is one of the commonest substances being misused [11], the present study aims at measuring the various aspects of burden on the caregivers or family members of alcohol dependent patients.

 Aims and objectives are as follows:

1. Assess the pattern of burden on the caregivers of patients with alcohol dependence syndrome.
2. Assess the relationship between the severity of dependence and the burden on caregivers.

## 2. Materials and Methods

### 2.1. Design

This cross-sectional descriptive study was conducted at the Department of Psychiatry, Sri Ramachandra Medical College and Research Institute, Porur, Chennai. Before the commencement of the study, the Institutional Ethical Committee (IEC) approval was obtained. Our IEC adheres to Indian Council of Medical Research (ICMR) guidelines for biomedical research in human beings.

### 2.2. Participants

The sample was comprised of 200 patients diagnosed with alcohol dependence syndrome and their 200 caregivers. The samples were inducted through consecutive sampling method.

### 2.3. Inclusion Criteria

The inclusion criteria were as follows: patients more than 18 years of age who were diagnosed to have alcohol dependence syndrome as per ICD-10 criteria and their caregivers who were more than 18 years of age. Patients and caregivers gave consent.

### 2.4. Exclusion Criteria

Patients and caregivers who had any other psychiatric comorbidity or those who are physically too ill to participate in the study were excluded. Caregivers with alcohol dependence and patients with any other dependence other than alcohol and nicotine were also excluded.

## 3. Assessments

The study was conducted over a period of one year from April 2015 to March 2016. Out of about 276 patients who were approached, 38 patients and caregivers did not fulfill the inclusion and exclusion criteria. 31 participants did not give consent to participate in the study. Seven participants gave consent but did not complete the study. Thus the final sample was comprised of 200 patients who were diagnosed to have alcohol dependence syndrome and their caregivers. The sociodemographic details were collected from both the patients and their caregivers. The severity of dependence was assessed using the Severity of Alcohol Dependence Questionnaire (SADQ) which has 20 items, each of which is scored on a scale of 0 to 3.

The caregivers of the patients were assessed using Family Burden Interview Schedule (FBIS). The FBIS is a semistructured interview schedule developed by ShailaPai and Kapur in 1981. It has 24 items each rated on a three-point scale: 0, no burden; 1. moderate burden; and 2, severe burden. This scale has been developed for the Indian setting, keeping in mind the socioeconomic and cultural conditions in India. The validity and reliability of the scale have been found to be satisfactory. The alpha coefficient of internal reliability of the FBIS was reported to be more than 0.78 by the authors, which indicates that the present schedule is a reliable tool [12]. During the development of the scale, the authors found their sick relatives experienced most burden on the family finances, the disruption of normal family activities, and production of stress related symptoms in family members due to patient illness [13].

On the basis of these findings, family burden has been assessed on six domains. (1) Financial burden has six items such as loss of patient's income and loss of income of other member of the family due to patient's illness. The scores range from 0 to 12: 0 to 2 no burden; 2 to 6 moderate burden; more than 6 severe burden. (2) Disruption of family routine activities has five items such as not going to work. The scores range from 0 to 10: 0 to 2 no burden; 2 to 5 moderate burden; more than 5 severe burden. (3) Disruption of family leisure has four items such as another family member's holiday or leisure time was used up by patient illness. The scores range from 0 to 8: 0 to 1 no burden; 1 to 4 moderate burden; more than 4 severe burden. (4) Disruption of family interaction has five items such as disruption of the general atmosphere in the house. The scores range from 0 to 10: 0 to 2 no burden; 2 to 5 moderate burden; more than 5 severe burden. (5) Effect on physical health of others has two items such as other family member suffered physical ill health and injuries due to patient's behavior. The scores range from 0 to 4: 0 no burden; 1 to 2 moderate burden; more than 2 severe burden. (6) Effect on mental health of others has two items such as psychological illness in other family member due to patient's behavior. The scores range from 0 to 4: 0 no burden; 1 to 2 moderate burden; more than 2 severe burden. The total score of these six domains is the objective burden ranges from 0 to 48. Each interview would last for about 15 to 20 minutes. The scores are from 0 to 6 no burden; 6 to 24 moderate burden; and above 24 severe burden.

A standard question to assess the “subjective” burden is also included in the schedule. This is to be assessed by asking the following standard question and scoring the relative's answer: How much would you say you have suffered owing to the patient's illness, severely, a little, or not at all.

## 4. Statistical Analysis

The data was analyzed using the Statistical Package for Social Scientists, version twenty (SPSS-20). Discrete variables were computed as frequency and percentage. Mean and standard deviation was calculated for all the continuous variables. Karl Pearson's correlation was used for computing correlations of parametric variables. Significance was compared using two-tailed *p* values. The significance level was set at <0.05.

## 5. Results

### 5.1. Demographic Characteristics of the Patients

As depicted in Table 1, in our study, all the 200 patients were males and most of the patients were in the fourth and fifth decade of life. Almost 3/4 of them were married and were living with their spouses. Fifty per cent of the population was educated up to higher secondary level and 15% were graduates. More than 3/4 of the patients were employed and only 23 were unemployed. More than half of the sample patients had nuclear families. Most of the patients hailed from urban background, belonging to middle socioeconomic status, and were Hindu by religion.

### 5.2. Demographic Characteristics of Caregivers

As depicted in Table 1, 180 of caregivers in our study were females and only 20 were males. Among 20 males, 18 were fathers and two were uncles. Among the female caregivers, 3/4 of them were spouses. 32% of the caregivers were either the mother or father. Siblings contributed to a very small proportion of caregivers (2.5%). When compared to the patients, a large number of caregivers were illiterates (21.5%) and most of them were unemployed. The religion, family type, socioeconomic status, and locality were on a par with the patients.

### 5.3. Clinical Characteristics and Dependence Pattern of the Patients

As shown in Table 2, (52%) 104 patients reported mild dependence, (31%) 62 patients moderate dependence, (15%) 30 patients severe dependence, and only (2%) 4 patients very severe dependence. The average score on SADQ was 19.62 ± 11.28. Nearly 80% of alcohol dependent patients were drinking minimum of 180 ml of Indian Made Foreign Liquor (IMFL) per day which contains about 76.5 ml of absolute alcohol. IMFL is a distilled spirit (i.e., brandy, whiskey, and rum). Our patients consumed predominantly brandy. Each 100 ml of IMFL contains 42.5% of absolute alcohol. Almost 40% of dependent patients were drinking around 360 ml of IMFL every day and 8% of dependent patients were drinking 750 ml of IMFL per day. Tremors were commonly noted in most of the patients.

## 6. Family Burden Interview Schedule

As depicted in Table 3, the total objective burden scores across all the domains was found to be 17.02 ± 10.56. Subjectively, 116 (58%) of the caregivers experienced severe burden and 73 (36.5%) had moderate burden. Thus the results showed that the caregivers had experienced moderate to severe burden.

5.5% of the caregivers reported as having no subjective burden. In the domain scores, more than half of the caregivers had experienced severe financial burden because of the loss of the patient's income. In the disruption of routine family activities domain, caregivers experienced moderate to severe burden because patients were not helping them in their household activities and patient's lack of attention to other family members. In the disruption of the family interaction domain, almost all caregivers experienced severe burden since nearly 80% of the patients were causing disruption in the general atmosphere of the house. About 3/4 of the caregivers reported having arguments related to alcohol use. In the effect on the physical health of others domain, almost all (95%) of the caregivers reported that there was no burden, whereas, in the effect on the mental health of others domain, 58% of the caregivers reported to have moderate to severe burden in mental well-being due to loss of sleep, irritability, depressed mood, and death wishes secondary to the patient's alcohol usage.

### 6.1. Correlation between Severity of the Dependence with Caregiver Burden

As depicted in Table 3, correlation between the severity of patient's dependence with their caregiver burden was statistically analyzed. The results showed that there was significant correlation of 0.67 at *p* value <0.001 between the severity of dependence and the total objective burden scores. Caregiver subjective burden score also significantly correlated at 0.48 with *p* value <0.001. In the domain scores, the correlation was strongest for financial burden compared to other domains with the severity of dependence at correlation coefficient of 0.66 with the level of significance <0.001. In addition, all the other domains in the FBIS also significantly correlated with the dependence severity.

## 7. Discussion

Alcohol dependence is a severe mental health problem associated with health issues and social and financial burden not only for the patient but also for the family members. In addition, it assumes greater relevance to predict the outcome of the alcoholism. Our study assessed the burden experienced by caregivers of treatment seeking alcohol dependent subjects. Of the 200 subjects in our study, all of them were males which show that in our centre mostly males seek deaddiction treatment which is same as in other part of country. Much of the study's sociodemographic profiles of the caregivers were matched with one similar study done in Ranchi, India [14], in the past. Majority of the caregivers were females; they were predominantly spouses of the patient. In a country like us, there is a cultural belief that men should be the breadwinner of the family and probably this would have shifted the responsibility of caring for the sick to the women [14]. A western study also reported that the female affected family members exceed male caregivers particularly partners were more than mothers and sisters. They also had significant male affected family members such as father, uncle and brothers who are slightly different from our study sample [15].

In India, unlike western population the people mostly live in joint families. Though our study samples living in nuclear families were slightly more than those living in joint families, the difference is less. However in our study the proportion of families living in joint family was much higher than the western population. In the joint family morbidity of patients could easily shift to their family members since everyone were exposed to the patient's alcohol related problems on a day to day basis. Their daily activities got disrupted frequently and all family members may get exposed to physical injuries due to violent behavior of patients under intoxicated state. In addition children in the family would have a poor role model by seeing the patient's behavior. Though most of our subjects came from urban background, majority of them belonged to lower class to lower middle income group. This is probably due to rapid expansion of the city with migrants from adjacent town.

In our study, the caregivers experienced significant burden in various domains due to patient's alcoholism. It is probably because the spouses were dependent on the patients for various reasons like finance and child-rearing. Moreover, the societal views of being separated from the husbands suffering from alcoholism will cause them more mental trauma and hence most of them chose to live with the patients even though they experienced significant burden. More than 3/4 of our caregivers were wives having children of varying age. Patient's dependence severity was positively correlated with their caregiver burden at the correlation coefficient value of 0.67 which means that the correlation was highly significant. The various domains such as financial burden, disruption of routine family activities, disruption of family interaction, effect on the physical health of others, and effect on the mental health of others were also positively correlated with highly significant correlation coefficient value. This is possibly due to the fact that, in most of the families, patients were the sole earning member of the family and majority of the caregivers were unemployed. Also money was deviated for procuring the substance and treatment expenditures [16]. Frequent arguments, verbal abuse, and physical abuse of family members under the influence of alcohol caused significant disruption in the communication between family members, disruption in their leisure activity, and significant adverse impact on caregiver physical and mental health.

## 8. Limitations

Though our study found significant caregiver burden, the following limitation has to be kept in mind while interpreting the results such as limited sample.

## 9. Conclusion

The present study found that there is significant burden for caregivers. In addition, the caregiver burden and severity of dependence were positively correlated with high level of significance. Therefore, while treating alcoholics, it is important to alleviate the burden of the caregivers which in turn will lead to better treatment effectiveness.

## Figures and Tables

**Table 1:** Sociodemographic profile of the patients and caregivers.

Sociodemographic variables	Patient *n* = 200, frequency (%) mean ± SD	Caregiver *n* = 200, frequency (%) mean ± SD
Age	38.73 ± 9.51	41.60 ± 14.45

Sex		
Male	200 (100%)	20 (10%)
Female		180 (90%)

Marital status		
Single	41 (20.5%)	3 (1.5%)
Married	153 (76.5%)	175 (87.5%)
Others	6 (3%)	22 (11%)

Education		
Illiterate	14 (7%)	43 (21.5%)
Primary school	31 (15.5%)	39 (19.5%)
Middle school	51 (25.5%)	34 (17%)
Higher secondary	73 (36.5%)	63 (31.5%)
Graduate	31 (15.5%)	21 (10.5%)

Occupation		
Unemployed	23 (11.5%)	166 (83%)
Unskilled labour	52 (26%)	10 (5%)
Skilled labour	85 (42.5%)	14 (7%)
Clerical/shop owner	32 (16%)	10 (5%)
Professional	8 (4%)	

Family		
Nuclear	112 (56%)	112 (56%)
Joint	87 (43.5%)	87 (43.5%)
Extended	1 (0.5%)	1 (0.5%)

Religion		
Hindu	171 (85.5%)	171 (85.5%)
Muslim	14 (7%)	14 (7%)
Christian	15 (7.5%)	15 (7.5%)
Others		

Income	10,649 ± 7151	7300 ± 4764

Socioeconomic status		
Upper	6 (3%)	7 (3.5%)
Upper middle	38 (19%)	38 (19%)
Lower middle	64 (32%)	64 (35%)
Upper lower	85 (42.5%)	84 (42%)
Lower	7 (3.5%)	7 (3.5%)

Locality		
Urban	134 (67%)	135 (67.5%)
Rural	66 (33%)	65 (32.5%)

Relationship of the caregiver with patient		
Parent		64 (32%)
Spouse		129 (64.5%)
Sibling		5 (2.5%)
Others		2 (1%)

**Table 2:** Classification of alcohol dependent patients based on severity.

Severity of dependence	SADQ total score	*n* = 200 (%)
Mild dependence	4–19	104 (52)
Moderate dependence	20–30	62 (31)
Severe dependence	31–44	30 (15)
Very severe dependence	≥45	4 (2)

**Table 3:** Correlation between the severity of dependence and the burden on the caregivers.

FBIS domain score	Mean ± SD	Correlation coefficient
Financial burden	4.56 ± 3.19	0.66^**∗∗**^
Disruption of routine family activities	4.67 ± 3.15	0.49^**∗∗**^
Disruption of family leisure	1.85 ± 2.17	0.48^**∗∗**^
Disruption of family interaction	4.54 ± 2.92	0.55^**∗∗**^
Effect on the physical health of others	0.33 ± 0.74	0.43^**∗∗**^
Effect on the mental health of others	1.08 ± 1.09	0.38^**∗∗**^
Objective burden	17.02 ± 10.56	0.66^**∗∗**^
Subjective burden	1.52 ± 0.6	0.47^**∗∗**^

Correlation is significant at the *p* value: *p* value **<0.005**^**∗∗**^ level (two-tailed).
